# Legal Environment, Technological Innovation, and Sustainable Economic Growth

**DOI:** 10.3389/fpsyg.2022.929359

**Published:** 2022-07-28

**Authors:** Yidan Zhao

**Affiliations:** Law School, Southwestern University of Finance and Economics, Chengdu, China

**Keywords:** legal environment, technological innovation, sustainable economic growth, intellectual property protection, legal system

## Abstract

The productivity gains generated by innovation are the root cause of long-term economic growth. In this paper, two empirical hypotheses are proposed to clarify our view: the trade turnover of technology market and intellectual property protection are important factors to stimulate innovation; The main channel of communication is through the increase of research staff and R&D funds. The empirical research result show that: (1) The greater the technology trade volume, the greater the incentive to regional innovation activities, the greater the number of regional patents; (2) About the intellectual property protection, The higher the protection intensity is, the greater the incentive is to regional innovation activities and the greater the number of regional patents. Different from relevant researches, this paper discusses the decisive role of market activity and legal environment on regional innovation behavior from the perspective of technology trade and patent protection, and emphasizes the theoretical significance of market and legal system from a more general perspective.

## Introduction

The productivity gains generated by innovation are the root cause of long-term economic growth. Schumpeter first studied the factors that influence firms’ innovation activities. He believes that innovation and R&D require capital scale, and only large enterprises can absorb the costs and losses of R&D. The subsequent researches mainly focus on market monopoly power and enterprise scale and analyze the conditions and incentives of innovation activities. In the “endogenous growth theory” of modern macroeconomics (Romer and Romer, 1989), the market power of innovators and the market scale they face play a core role in promoting innovation activities (Cheng and Dinopoulos, 1992).

The researchers of endogenous growth theory emphasize the organization and demand factors of innovation activities such as market size and market power. In contrast, researchers of institutional economics focus on the supply side, arguing that institutional factors – the environment that encourages market-based technological products and legal protection of intellectual property rights – play a key role in motivating innovators to carry out technological innovation. North believed that the intellectual property protection system with patent system as the core played an important role in the emergence of industrial revolution and the rise of Western countries (North, 1990). Acemoglu and Robinson (2019) developed this view, believing that the extensive system environment established by the British “Glorious Revolution” constituted the property right protection of innovation activities, induced innovation activities to occur continuously, and enabled more people to devote their talents to commercial activities, leading to the industrial revolution. Existing theories generally only emphasize one factor that affects innovation activities, but the research on the factors that affect supply and demand of innovation activities is insufficient. This paper attempts to quantitatively examine the impact of external market demand and property right protection environment on China’s regional innovation and its transmission mechanism.

There is much room for improvement in China’s innovation capability at the present stage. China’s economic growth is mainly concentrated in labor-intensive industries, the overall economy is highly dependent on exports, and industries with a large proportion are at the bottom of the industrial chain, without obvious innovation advantages and enthusiasm (Zhang et al., 2007). In addition, there are problems such as weak support for innovation achievements, insufficient innovation capacity of enterprises, and lack of high-end innovative talents. China not only needs to strengthen the investment of personnel and funds, but also needs to systematically study the factors affecting enterprises’ innovation activities, which is of great significance to encourage enterprises’ independent research and development, build an innovative country, and realize the transformation and upgrading of China’s industrial structure.

From the perspective of literatures development, the research on innovation activities mainly tests the relationship between firm scale, market power, innovation input, and innovation output. Horowitz (1962) and Hamberg (1964) provide evidence that enterprise size and market power significantly affect R&D investment. Subsequent studies have paid attention to the impact of production technology on innovation. Technological differences reflect the characteristics of industries and directly lead to different effects of factors affecting innovation behavior. These studies mostly combine technological difference with market concentration and believe that market force is the main factor affecting R&D and innovation investment. Typical articles include Comanor (Schwartz, 1975), etc. Other researchers believe that the impact of firm size and market power on innovation is not a simple linear relationship, but an “inverted U-shaped” relationship (Zhang et al., 2007).

The existing literatures mainly divide the factors that affect the innovation behavior into two categories: the internal structure of enterprises and the external environment of enterprises. Literatures on enterprise internal structure mainly emphasize the influence of enterprise characteristics on innovation behavior, such as enterprise size (Huang and Chen, 2011), industry chain positioning, ownership structure (Wu, 2009), salary incentive mechanism (Chen et al., 2011), enterprise sales revenue (Wu, 2009), enterprise human capital structure, etc. The literatures related to enterprise external environment mainly focus on the incentive of regional market and industrial structure on innovation activities, including financial development degree and financing environment (Wei et al., 2007), industrial division of labor (Zhang et al., 2007), foreign investment and technology transfer (Pan and Yu, 2009), Government R&D funding (Hu, 2001), and social capital (Ping et al., 2013). These studies examine the factors that influence regional innovation from many aspects, but largely ignore the institutional factors that stimulate innovation.

As for technological innovation and foreign trade, as early as the beginning of the 20th century, since Schumpeter put forward the theory of technological innovation, scholars of various countries have been studying technological innovation in the ascends. Scholars have done a lot of research on how developing countries promote their own technological progress and innovation through foreign trade. In the middle and late 1980s, the new growth theory represented by Romer, Lucas and others gradually replaced the traditional development economics, because it emphasized the importance of technological progress for economic development, and further internalized technology, thus establishing the endogenous growth theory. Endogenous growth theory attaches great importance to the spillover effects of knowledge and technology acquired by developing countries from international trade. Grossman and Helpman (1991) believed that international trade was the engine of national technological progress and innovation. Coe et al. (1997) analyzed the influence and approach of international trade on promoting technological progress, and believed that international trade gave technologically backward countries an opportunity to carry out technological imitation, and technological imitation was A “dry learning” technological progress process proposed by A DAM V: International trade not only improves the labor productivity of trading countries, improves the efficiency of RESEARCH and development activities of trading countries and avoids repetitive labor, but also indirectly affects the progress and cost of technological innovation by influencing the international factor market, so as to achieve the goal of improving the technological innovation level of trading countries. Coe and Helpman (1995) and Coe et al. (1997) also confirmed through a large number of empirical studies that international trade can significantly improve TFP (total factor productivity) and economic growth of trading countries.

In terms of technological innovation and human capital, human capital is an important channel for host countries to absorb international knowledge and technology spillover effect to improve their own technological innovation ability. Scholars of various countries have done a lot of research on the relationship between human capital and technological innovation. Borensztein et al. (1998) believed that the production of high-tech products in developed countries would help their human capital to improve their technological innovation ability through “learning through work.” Investigated the technology spillover effect of OECD countries on developing countries (Borensztein et al., 1998), and the study showed that human capital investment of a country was important for technology absorption and innovation. Foster’s research also shows that the increase of human capital stock is conducive to improving the technology absorption capacity of developing countries, thus enhancing the technological innovation capacity (Foster and Rosenzweig, 2003).

In terms of technological innovation and FDI, there have been abundant academic studies on the knowledge and technology spillover effects of FDI to improve the technological progress and innovation capacity of host countries. Mundell (1957) explained the substitution relationship between trade and investment from a relatively static perspective, that is, in the case of trade barriers hindering trade, FDI will take place as a kind of market entry in international trade. Development economists represented by Solow also studied this earlier. However, due to its high emphasis on the role of capital accumulation in economic growth, countries did not achieve significant results in practical application after world War II. In the 1960s, analyzed technology spillover effect as an important phenomenon of FOREIGN direct investment for the first time (Mcdougall, 1960). However, the new growth theory of the 1980s holds that the technological spillover effect of economic opening up, international capital flow and international trade accelerates the transfer of advanced science and technology, knowledge, and human capital around the world. The new growth theory takes technological factors as the core variable and discusses the impact of FDI on technological innovation and economic growth in the host country from the perspective of technological progress and technological spillover. This idea has triggered many scholars’ empirical tests on the hypothesis of FDI technological spillover makes use of cross-section data of Australian industries in 1969 to make a quantitative analysis of the influence of foreign capital on local labor productivity, and finds that FDI does have a positive impact on labor productivity of relevant industries in Australia (Caves, 1974). Examined the impact of Japanese investment into the United States and found. The inflow of Japanese capital not only promotes the improvement of production efficiency of local Enterprises in the United States, but also improves the technological innovation level of Japanese enterprises in the United States (Lee and Barro, 2000). However, the situation of developing countries and regions is more complicated, and the relevant empirical studies on FDI lack consistent conclusions. Such as Kokko (1996) found significant positive FDI technology spillover effect in Mexico, Indonesia (Blomstrom and Sjoholm, 1999; Blalock and Gertler, 2003). Accordingly, investigated the panel data of Moroccan manufacturing industry from 1985 to 1989 and found no positive spillover effect. Haddad and Harrison (1993) examined the panel data of Venezuelan enterprises from 1976 to 1989 and found that FDI was negatively correlated with TFP of domestic factories (Aitken and Harrison, 1999).

The internal structure and external market environment of an enterprise determine its innovation ability and demand for innovation activities, but only a perfect legal environment can ensure that innovators can get benefits from innovation activities. A few literatures have studied the influence of institutional factors on Innovation activities in China. Zhu (2004) used the output competition model of oligopoly to find that, considering the introduction of foreign capital, strengthening intellectual property protection can significantly improve the welfare of developing countries. Researcher believe that effective implementation of better intellectual property protection will improve enterprises’ ability to obtain external financing and make innovative investment. Researchers uses a natural experimental study to find that the introduction of low-cost fakes leads to the improvement of product quality and price of genuine production enterprises. In general, although the above studies provide evidence of the different impacts of institutional environment on enterprise innovation behavior from the micro perspective, the research samples are mainly limited to certain specific industries, and relevant research conclusions are difficult to be extended to the overall level of the region.

## Research Design

### Empirical Hypothesis

In this paper, two empirical hypotheses are proposed to clarify our view: the trade turnover of technology market and intellectual property protection are important factors to stimulate innovation; The main channel of communication is through the increase of research staff and R&D funds.

In an open market environment, technology market has realized the transformation from scientific research achievements to product production. Technology transfer directly profits knowledge products and brings rich returns to R&D subjects. The activity of technology transfer markets can stimulate regional innovation in two ways. On the one hand, the income from the transfer and sale of scientific and technological products transmits observable market demand signals to other subjects, and the high technology transaction fees encourage other individuals to engage in innovative behaviors in order to gain benefits through technology transaction, which is the “external demonstration effect” of technology transaction. On the other hand, the purchase of new technological products provides opportunities for imitation and improvement. Through the learning of knowledge products, the corresponding improvement can be made to develop products with more market value. This optimization of internal resources is called the “internal demonstration effect” of technology trading.

The protection of the legitimate rights and interests of technology developers also determines the intensity of regional innovation activities. If the protection of innovative activities is not enough, the products developed by innovative entities that invest a lot of money and personnel will be easily copied, which inhibits further innovation activities and reduces the enthusiasm of the whole society for innovation. Therefore, the protection of intellectual property rights and developers’ rights and interests in the market protects the legitimate rights and interests of innovative main products, encourages more innovative behaviors of innovative entities, and also encourages other entities to join innovative activities.

From the perspective of “demonstration effect” and “protection effect,” this paper propose the following hypotheses to be tested:

Hypothesis 1a: Technology market transaction volume has a “demonstration effect” on regional innovation activities. The greater the technology trade volume, the greater the incentive to regional innovation activities, the greater the number of regional patents.
Hypothesis 1b: Intellectual property protection has a “protection effect” on regional innovation activities. The higher the protection intensity is, the greater the incentive is to regional innovation activities and the greater the number of regional patents.

The second empirical hypothesis to be tested in this paper mainly verifies the transmission mechanism of “demonstration effect” and “protection effect” from the perspective of innovation input:

Hypothesis 2: The “demonstration effect” of technological market turnover and the “protection effect” of intellectual property protection stimulate regional innovation activities by increasing regional scientific research personnel and investment.

### Econometric Model

In this paper, the number of patented products applied in each region is used to measure the innovation situation of the region, and the transmission mechanism is analyzed by using the scientific research practitioners and R&D funds in the region. The following measurement model is used in this paper:


(1) $$\begin{array}{c} {\text{patent}}_{i,t}=\alpha_{0}+\alpha_{1}{\text{mkt\_value}}_{i,t-1}+\alpha_{2}{\text{protect}}_{i,t-1}+\varphi {\text{X}}_{i,t-1}\\ \;+\sum {\text{provin}}_{\text{i}}+\sum {\text{year}}_{\text{t}}+{\text{u}}_{i,t} \end{array}$$

In Model 1, patent is the explained variable of the model to measure the innovation degree of each region. mkt_value and protect are the main explanatory variables. As the measurement indicators of “demonstration effect” of technology market and “protection effect” of intellectual property, X represents other control variables of the model. Province fixed effect *provin* and time effect year are also controlled. Model 1 was mainly used to test hypothesis 1a and 1b, and the coefficients of mkt_value and protect were significantly positive.

The following model is used to test hypothesis 2 and analyze the channels through which “demonstration effect” and “protection effect” stimulate regional innovation activities.


(2) $$\begin{array}{c} {\text{rd\_input}}_{i,t}=\beta_{0}+\beta_{1}{\text{mkt\_value}}_{i,t-1}+\beta_{2}{\text{protect}}_{i,t-1}+\varphi {\text{Z}}_{i,t-1}\\ \;+\sum {\text{provin}}_{\text{i}}+\sum {\text{year}}_{\text{t}}+{\text{u}}_{i,t} \end{array}$$

where, rd_input is the explained variable, representing the regional R&D investment. R&D investment is multi-dimensional, including the number of research institutions, researchers, universities, high-tech enterprises, R&D investment, and other indicators, taking into account the availability of research data, this paper mainly measures regional R&D investment from human and financial input.

### Index Selection and Data Sources

Indicators in this paper are mainly composed of three parts: variables related to the degree of innovation, variables related to the marketization of technological products and patent protection, and control variables. The data of 283 prefecture-level cities from 2001 to 2011 are used as samples for analysis.

Variables related to the degree of innovation: This index is mainly divided into two parts. The first part is the number of authorized patents applied for three patents (invention patents, practical patents, and design patents) in prefecture-level cities. This data is summarized according to the number of patents in each region each year in the patent retrieval system. The other part is the number of scientific researchers rd_worker and the investment of scientific research funds rd_funding in each province, these data are from China Statistical Yearbook.

Main explanatory variables: This paper uses two explanatory variables, the amount of technology market transaction mkt_value and patent product protection protect. The transaction data of technology market comes from China Statistical Yearbook of Science and Technology. The logarithm of transaction amount of unit transaction is taken as the measurement index. The larger the transaction amount of unit transaction is, the higher the remuneration of innovation activities will be, and the more obvious the demonstration effect will be. And the number of settled cases of infringement disputes over the protection of patented products.

Select the following indicators as control variables:

Number of industrial enterprises above designated size firmnumber : enterprises above designated size have more capital and ability to carry out R&D. The more enterprises above designated size, the larger the enterprise base and the more regional innovation behaviors. Number of college students student_college : The larger the size of the college is, the more technical talents are reserved, and the greater the support for innovation talents is, and the more obvious the positive impact on innovation behavior is. Personnel employed in scientific research institutions science_worker : The personnel employed in scientific research institutions mainly describe the scale of scientific research, which can better reflect the regional differences in scientific research strength. Activity degree of commodity trading retail : measured by the logarithm of total retail commodities in the market, the more vigorous the demand for products and the supply of commodities in the region with intense market activities, the more vigorous the activity of commodity supply is, the more it promotes the emergence of new products. In addition, regional economic development level (per capita GDP), industrial structure first2gdp, second2gdp(output ratio of primary and secondary industries), and population density density are used as control variables of the model.

In the regression model, the main variables are logarithmic processing; Considering the time delay, the main explanatory variables and control variables in models 1 and 2 lag behind one period.

### Data Description

Table 1 is the descriptive statistics of the main variables. It is not difficult to find that the sample cities have significant differences in patent application authorization, patent product protection, the number of researchers and other aspects, and the gap between the maximum and minimum value of technological market transaction volume and scientific research expenditure are also obvious.

**TABLE 1:** Descriptive statistics of main variables.

Variable	Variable declaration	Observations	Mean value	Standard deviation	Minimum value	Maximum value
patent	Number of patent Applications authorized (pieces)	3,091	1009.42	3544.1	0.000	89768
mkt_value	Technology market unit turnover (10,000 yuan/piece)	3,091	72.077	52.662	4.441	386.387
protect	Number of infringement disputes (cases)	3,089	43.573	59.809	0.000	314.000
rd_worker	Regional scientific research personnel (10,000 people)	3,091	6.736	6.783	0.080	41.080
rd_funding	Regional research expenditure (100 million yuan)	3,091	4.294	1.214	0.588	6.972
firmnumber	Number of enterprises above designated size	3,065	940.494	1397.956	19.000	13720
student_college	Average number of students per university (10,000 people)	2,856	0.817	0.465	0.014	4.900
science_worker	Personnel of scientific research institutions (10,000 people)	3,091	1.970	1.341	0.081	6.930
retail	Total Retail Sales of Consumer Goods (100 million yuan)	3,063	163.611	324.227	1.462	4964.000
first2gdp	Primary Industry Output/GDP(%)	3,068	18.593	12.398	0.060	88.690
second2gdp	Secondary Industry Output/GDP(%)	3,068	48.255	11.713	9.000	91.470
gdppercap	Log (GDP per capita)	3,069	9.592	0.843	7.401	12.970
density	Population density (thousand people/square kilometers)	3,069	0.466	0.510	0.005	11.564

*(1) Staff of scientific research institutions mainly refer to staff engaged in scientific research, excluding administrative staff of scientific research institutions. (2) In the following regression, any variable (x) is converted into l(x), indicating logarithmic processing.*

## Empirical Analysis

### “Demonstration Effect” and “Protection Effect”: Basic Models

Table 2 shows the OLS estimation results of model 1. Column 1 only tests the results of “demonstration effect,” and the regression coefficient shows that hypothesis 1a is supported: the “demonstration effect” of technology market transaction on regional innovation is obvious: the single technology market transaction value increases by 10%, and the number of regional patents increases by 0.88%. The regression coefficient of firmnumber is significantly positive, which verifies the traditional theory that firm’s scale effect can promote regional innovation. From the *R*^2^ coefficient, the benchmark model can explain 3/4 of the variance of regional innovation activities. This indicates that “demonstration effect” and “protection effect” are important factors affecting regional innovation level.

**TABLE 2:** Demonstration effect and protection effect: test results of hypotheses 1a and 1b.

	(1)	(2)	(3)	(4)	(5)
L.lmkt_value	0.0854*** (0.0319)		0.0864*** (0.0314)	0.0912*** (0.0327)	0.0889*** (0.0328)
L.protect		0.0659*** (0.0157)	0.0673*** (0.0159)	0.0532*** (0.0151)	0.0494*** (0.0153)
L.lfirmnumber	0.1892*** (0.0675)	0.1630** (0.0671)	0.1507** (0.0659)	0.1581** (0.0684)	0.1374** (0.0695)
L.student_college				0.1409*** (0.0412)	0.1377*** (0.0408)
L.lsience_worker				1.0647*** (0.2158)	1.0955*** (0.2140)
L.lretail				0.1612*** (0.0571)	0.1375** (0.0560)
L.first2gdp					−0.0035* (0.0018)
L.second2gdp					0.0013 (0.0035)
L.gdppercap					0.0743 (0.1156)
L.density					−0.0464* (0.0237)
The number of observations	2,774	2,772	2,772	2,568	2,566
*R* ^2^	0.756	0.758	0.759	0.789	0.790

*, **, and *** represent significance levels of 10, 5, and 1%, respectively. Robust standard error in parentheses. The constant term omitted unreported, controlled year and fixed effect of province, as shown in the following table.

Column 2 only tests whether the “protection” of patented products promotes innovation behavior, and it can be found that hypothesis 1b is also verified: every 10% increase in patent protection measured by the number of infringement cases, the increase of patented products is about 0.66%. In the third column, both “demonstration effect” and “protection effect” are analyzed. The coefficient and significance of technical product marketization index and patent protection index are added into the regression model at the same time. The fourth column controls the average number of students in each university, the number of researchers engaged in scientific research and the retail sales of consumer goods as control variables. The results show that the three factors all positively affect the regional innovation level, and the regression coefficients are 0.14, 1.06, and 0.16, respectively, and the influence coefficient of the size of the research staff is the largest. After controlling for all three factors, hypothesis 1 still holds.

The last column of Table 2 also controls the per capita GDP of each city, the output share of primary and secondary industries, and the regional population density. The results show that the scale of primary industry has a negative impact on innovation behavior: when the proportion of agricultural economy increases by 1%, the number of patent applications will decrease by 0.3%. This may be because agricultural production is still in traditional ways and the demand for new technologies is not high, which stifles innovation. But even after controlling for economic size and structure, the “demonstration effect” and “protection effect” remain.

### Robustness Test

In order to prove that the hypothesis in this paper is not limited to specific explained variables, robustness tests are performed in this section.

Firstly, two sub-indexes of “China Marketization Index,” less_protect and legal_protect are used as the substitute indexes of “demonstration effect” and “protection effect.” “Reducing local protection of goods” is measured by the ratio of trade protection to GDP encountered by a sample of firms selling goods across the country. The lower the ratio, the weaker the local protectionism. In regions with serious regional protectionism, enterprises can get “special attention” from the government, which reduces the enthusiasm of R&D and market sensitivity, and the “demonstration effect” generated by technology exchange is weak. In the sample of this paper, the correlation coefficient between the index of “reducing local protection of goods” and the turnover of technology market is 0.29, which is significant at the level of 1%.

The first column of Table 3 analyzes the impact of “reducing local protection of commodities” on innovation; the second column adds indicators of “protection effect”; the third and fourth columns add relevant control variables such as enterprise scale, scientific research investment, industrial structure, economic scale, and population density. As can be seen from the regression results, the hypothesis of this paper is still valid if the index of “reducing local commodity protection” is used to describe the “demonstration effect.” The last three columns of Table 3 measure the “protection effect” with the indicator of “protection of legitimate rights and interests of producers,” which is obtained based on the legal environment information about “protection of legitimate business activities of enterprises” provided by the sampled enterprises. From the regression results, the previous research conclusions are still valid after the new index is used to measure the “protection effect.”

**TABLE 3:** Robustness test: by changing different main explanatory variables.

	(1)	(2)	(3)	(4)	(5)	(6)
L.less_product	0.0759*** (0.0156)	0.0731*** (0.0155)	0.0660*** (0.0151)			
L.legal_protect				0.0522*** (0.0144)	0.0549*** (0.0144)	0.0466*** (0.0139)
L.protect		0.0586*** (0.0155)	0.0387*** (0.0149)			
L.lmkt_value					0.0512 (0.0319)	0.0564* (0.0337)
Other control variable	No	No	Yes	No	No	Yes
The number of observations	2,493	2,493	2,304	2,493	2,493	2,304
*R* ^2^	0.696	0.699	0.734	0.695	0.695	0.732

* and *** represent significance levels of 10 and 1%, respectively.

Furthermore, we control the influence of the following factors successively and observe the estimated coefficient of variables: (1) Openness level (openness), measured by the ratio of total import and export trade to GDP, the results show that the hypothesis that openness promotes regional innovation is confirmed; Financial development (finan_comp) helps relieve financial pressure and reduce capital constraints of enterprises, according to the Chinese marketization index, which measures the level of financial development. Regions with higher levels of financial competition have higher levels of innovation. (3) Labor mobility (*Labormobility*), human capital has a significant impact on the R&D decisions of enterprises and individuals. The mobility of labor market determines whether the market can allocate high-tech talents to meet the demand of enterprises for innovative human capital. The results show that the higher the mobility of human capital, the more obvious the promotion effect of innovation behavior. (4) Foreign direct investment (measured by the ratio of foreign direct investment to GDP and fixed asset investment), competition effect, technology imitation effect and diffusion effect of talent flow brought by FDI will promote enterprises to innovate. The results show that FDI has a significant positive impact on regional innovation activities. (5) Scale of state-owned enterprises (lsoe_number): state-owned enterprises’ output to GDP (soe_output_ratio), and state-owned enterprises’ asset scale to GDP (soe_asset_ratio) are used to measure the scale of state-owned enterprises. As the direct main body of government control, state-owned enterprises can alleviate the market failure caused by incomplete exclusivity of knowledge production, thus leading to more innovative behaviors of state-owned enterprises. The results confirm the positive relationship between state-owned enterprises and innovation degree. On the basis of comprehensive consideration of the above factors, the coefficient signs and significance of lmkt_value and protect do not change substantially, and the conclusion above is still valid.

### Influence Channels of “Demonstration Effect” and “Protection Effect”

This section uses model 2 to test hypothesis 2. The first four columns of Table 4 describe the impact of “demonstration effect” and “protection effect” on regional researchers. Column 1 and 2, respectively investigate the influence of transaction volume of technology market and number of infringement cases settled on the number of scientific research personnel. In Column 3, the above two factors are added simultaneously, and other relevant control variables are added in column 4. According to the regression results of the first four columns, both “demonstration effect” and “protection effect” significantly promote the increase of the number of researchers engaged in scientific research, and the number of researchers engaged in scientific research increases by about 50 and 0.24%, respectively when the transaction volume of unit technology market increases by 10% and the protection intensity of patent product increases by 10%. The last four columns of Table 4 test the influence of “demonstration effect” and “protection effect” on regional scientific research investment, and the structure of the regression model is similar to the first four columns. According to the regression results, “demonstration effect” and “protection effect” also have a positive promoting effect on the scientific research fund investment of scientific research institutions, and the scientific research fund increases by about 50 and 0.38%, respectively when the market turnover of each technology increases by 10% and the protection intensity of patent products increases by 10%. The “demonstrative effect” has a similar influence on the expenditure of scientific research personnel and the expenditure of scientific research funds, while the “protective effect” has a significantly greater influence on the expenditure of scientific research personnel.

**TABLE 4:** Input of scientific researchers and research funds.

	Y = lrd_worker				Y = lrd_funding			
	(1)	(2)	(3)	(4)	(5)	(6)	(7)	(8)
L.lmkt_value	0.0504*** (0.0123)		0.0513*** (0.0117)	0.0554*** (0.0123)	0.0456*** (0.0118)		0.0478*** (0.0111)	0.0524*** (0.0117)
L.protect		0.0204*** (0.0055)	0.0248*** (0.0056)	0.0241*** (0.0059)		0.0377*** (0.0060)	0.0384*** (0.0061)	0.0376*** (0.0058)
L.lfirmnumber	0.1492*** (0.0261)	0.1434*** (0.0269)	0.1360*** (0.0272)	0.1506*** (0.0270)	0.1179*** (0.0242)	0.1066*** (0.0243)	0.0997*** (0.0245)	0.0829*** (0.0248)
					0.0512 (0.0319)	0.0564* (0.0337)		
Other control variable	No	No	No	Yes	No	No	No	Yes
Province fixed effect	Yes	Yes	Yes	Yes	Yes	Yes	Yes	Yes
Year fixed effect	Yes	Yes	Yes	Yes	Yes	Yes	Yes	Yes
The number of observations	2,774	2,772	2,772	2,566	2,774	2,772	2,772	2,566
*R* ^2^	0.798	0.797	0.800	0.818	0.962	0.962	0.963	0.966

* and *** represent significance levels of 10 and 1%, respectively.

## Conclusion

This paper mainly discusses the main factors influencing innovation in China. We believe that the “demonstration effect” based on technology market exchange and the protection effect based on intellectual property protection are the important factors determining regional innovation. The “demonstration effect” conveys the relevant information of market demand for innovation activities, which is manifested by the obvious “demonstration” effect of technology market transaction on innovation behavior, encouraging more innovation behavior; “Protection effect” is an important institutional factor to encourage individuals to engage in innovative activities and protect their legitimate intellectual property rights and innovation enthusiasm. At the same time, it is found that the main transmission mechanism of demonstration effect and protection effect is the increase of researchers and R&D funds. It is robust and consistent to conclude that technology market transactions and patent protection encourage individuals and firms to increase R&D investment, which leads to more innovation. Different from relevant researches, this paper discusses the decisive role of market activity and legal environment on regional innovation behavior from the perspective of technology trade and patent protection, and emphasizes the theoretical significance of market and legal system from a more general perspective.

It should be pointed out that China has made remarkable progress in improving intellectual property laws and regulations and strengthening patent protection, which has had a positive impact on promoting regional innovation activities. However, there is still room for further improvement in the construction of legal environment at the present stage, the awareness of property rights protection of enterprises and the public needs to be further improved, and the development of new technology also brings some challenges to the protection of intellectual property rights. Although China has made great strides in technology trading market development, technology trading market still exist in information system construction lag, the management is not standard, the scientific research project evaluation system is not sound, patent technology problems such as difficult to match supply with demand, this will undoubtedly restricted technology trading market in promoting the positive role of diffusion of innovation. Therefore, the policy meaning of this paper is to encourage the development of investment must be diversified, in every link of “production, study, and research” collaborative innovation, on the basis of promoting patent protection system gradually improve, to build a mature technology trading market operation model. At the same time, play technology trading market “demonstration effect” and “protective effect” of intellectual property rights protection system, is to encourage social innovation activities, and it is an effective way to promote the construction of national innovation system and realize the transformation and upgrading of Chinese industry.

## Data Availability Statement

The datasets presented in this article are not readily available because the data can be obtained by contacting the corresponding author. Requests to access the datasets should be directed to YZ, lizw@swufe.edu.cn.

## Author Contributions

The author confirms being the sole contributor of this work and has approved it for publication.

## Conflict of Interest

The author declares that the research was conducted in the absence of any commercial or financial relationships that could be construed as a potential conflict of interest.

## Publisher’s Note

All claims expressed in this article are solely those of the authors and do not necessarily represent those of their affiliated organizations, or those of the publisher, the editors and the reviewers. Any product that may be evaluated in this article, or claim that may be made by its manufacturer, is not guaranteed or endorsed by the publisher.
